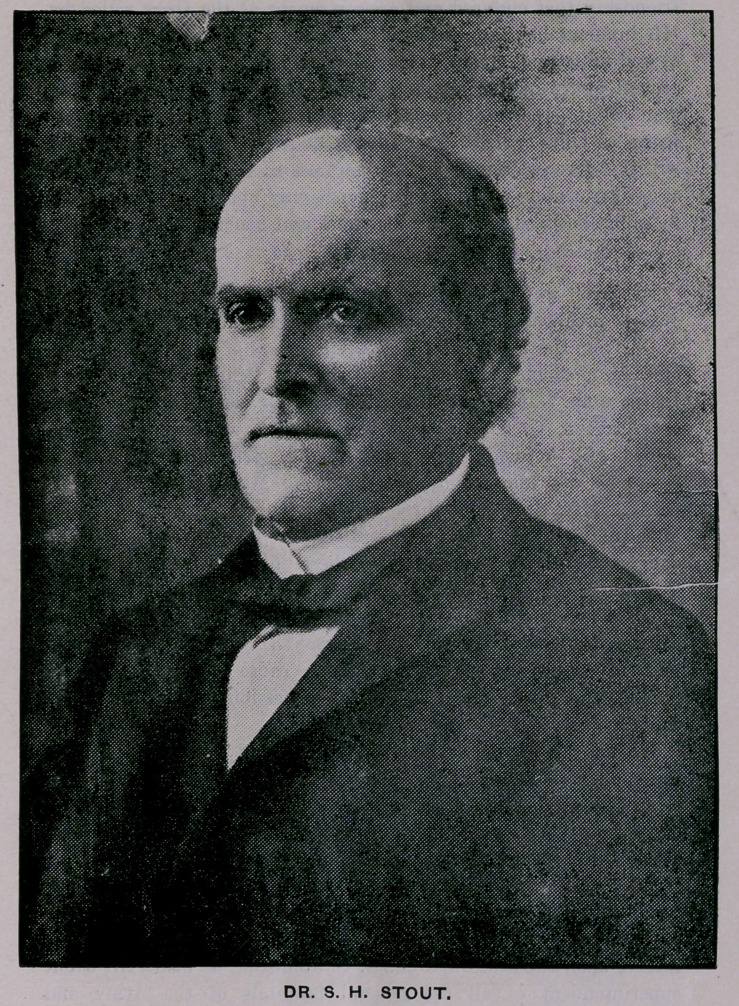# Samuel Hollingsworth Stout, A. M., M. D., LL. D.

**Published:** 1903-10

**Authors:** 


### TEXAS MEDICAL JOURNAL.

AUSTIN, TEXAS.

A MONTHLY JOURNAL OF MEDICINE AND SURGERY.

EDITED AND PUBLISHED BY
F. E. DANIEL, M. D.

ASSOCIATE EDITORS:

WITTEN BOOTH RUSS, M. D., San Antonio, Texas.

P. M. PAYNE, M. D., Brownwood, Texas.

Published Monthly at Austin, Texas. Subscription price $1.00 a year in advance.

Eastern Representative: John Guy Monihan, St. Paul Building, 220 Broadway, New York City.

Official organ of the the West Texas Medical Association, the Houston District Medical Association, the Austin District Medical Society, the Brazos Valley Medical Association, the Galveston County Medical Society, and several others.

Samuel Hollingsworth Stout, A. M., M. D., LL. D., Clarendon, Texas, late of Dallas, died at his home in Clarendon, September 18, 1903, in the 82nd year of his age.

Dr. Stout was known all over the civilized world as the organizer and Medical Director of the Hospital Department of the Confederate Army, in Tennessee, Mississippi, Georgia, Alabama and Louisiana. The most remarkable feature of the department was its mobilization—something unprecedented in history,—a feat that challenged the admiration of the world and drew forth words of praise and commendation not only from our own Confederate generals but from those of the other side, and even from Europe.

Dr. Stout was also extensively known as a scholar, an educator, a teacher and a surgeon. In his youth he had the advantages of a thorough classical education and was reputed to be one of the best Latin and Greek scholars in America.

As a man he was immensely popular and greatly beloved by all who knew him. A scholar, whose conversation was at all times entertaining and instructive, an optimist who never knew the meaning of the word "fail," a companion whose society was a delight and whose geniality and eternal youth were stimulating and inspir-

DR. S. H. STOUT.

ing. As father, husband, friend, citizen, physician, teacher,—in every relation of life, he was admirable. The writer knew him intimately, served with him in the trying times when was begun a friendship that endured and grew stronger as the years rolled by. His innumerable host of friends all over America will grieve with us and unite to do honor to his memory. He was the foremost and central figure in the famous Hospital Department of the Confederate States, the most distinguished, around which were grouped scores of able, brilliant and since famous surgeons. His memory will live forever, for he left the impress of his strong personality and vigorous and original thought not only upon every community in which he lived, but upon his times and generations.

BIOGRAPHICAL.

Dr. Stout was born in Nashville, Tennessee, March 3, 1822, and grew to manhood and received his education in that city; graduated A. B. from the University of Nashville, class of 1839. He graduated in medicine from the University of Pennsylvania in 1848. In the same year he passed examination for commission as Assistant Surgeon, U. S. N., but as the Mexican war was over he resigned, and, returning to Tennessee, he settled in Giles county, where he was married the same year to Miss Martha M. Abernathy. Mrs. Stout and three children survive the doctor, Misses Margaret and Kate, and a son living in Atlanta, Ga. The two daughters are well known as teachers in the public schools of Texas. The University of Nashville (his *alma mater* in the arts), in 1885—forty-six years after giving him the degree of A. B.—conferred upon Dr. Stout the degree LL. D., "in acknowledgment of his distinguished services in the cause of humanity, in the pursuit and practice of the medical profession, and as an educator."

He was practicing medicine in Giles county when the war between the States broke out. He immediately entered the Confederate States service as surgeon of Colonel (afterwards Major-General) Jno. C. Brown's Third Tennessee Regiment: In October, 1861, was surgeon in charge of Gordon Hospital, Nashville. After the fall of Fort Donaldson (April, 1862), he was made "post surgeon" at Chattanooga, in charge of all the Confederate hospitals at that post. When Bragg took command of the Army of Tennessee, upon the death of Gen. A. S. Johnston, he made Dr. Stout superintendent of all the general hospitals of the Army and Department of Tennessee; and in February, 1863, the office of medical director of hospitals was created, and Dr. Stout was appointed by the Secretary of War to the position and filled it till the close of the war; reporting directly to the surgeon-general at Richmond. By the exigencies of the service before the close of the war, the territorial limits of the department were vastly extended, till it embraced everything from the Savannah river to the Mississippi. The hospitals in this vast area of country, invaded often and at many points by the enemy, were so "mobilized" that organizations were never broken, but the staff of each, together with its nurses, stewards, commissary, hospital furniture and all equipment, advanced or retreated, according as the army advanced or retreated, and often "got up and got out of the way" when threatened with capture. It was a wonder. Its movements were accomplished from point to point with as little confusion as were those of the army in front.

Dr. Stout preserved to the day of his death all the records, roster of officers, orders, and other documents of his department, and intended to write a full history of it, but never did; that is, he had written twenty-four chapters of it, under the head of "Records, Recollections and Reminiscences," etc., and they were published in the *Southern Practitioner* (Nashville), the official organ of the United Confederate Veterans' Association. Amongst these papers are the personal and official correspondence with General Bragg and other commanders, who highly praised and complimented Dr. Stout upon the efficiency of his department. I regret I have not room to publish some of these letters. It was General Bragg's intention, had he lived, to assist Dr. Stout to publish these valuable documents under the auspices of the Southern Historical Society, of which General Bragg was president. The Misses Stout hope to complete the work Dr. Stout had begun, and we hope to see the series in the *Southern Practitioner* unbroken.

After the close of the war Dr. Stout settled in Atlanta, Ga., where he was elected to a chair in the medical college (1866-7), but after two courses of lectures he resigned and returned to Giles county, Tennessee, to gather up the wreckage of his once ample fortune. He returned to Atlanta in 1869. In 1882 he removed to Texas, settling in Cisco; thence he removed, in 1893, to Dallas; thence to Clarendon in June, 1903. In Atlanta in 1869 he inaugurated the movement to establish the system of public schools of which that city now so justly boasts, and in Cisco, Texas, he organized the public schools, now so popular and successful.

In Dallas, up to the date of his removal to Clarendon, Dr. Stout was connected with the organization of the first medical college in that city—Medical Department of the University of Dallas—

and was Dean of the Faculty. At the time of his death he was Emeritus Professor of Medicine in that institution, now the Medical Department of Baylor University.

The remains of the noble and distinguished Confederate Veteran Surgeon were interred, with Masonic honors, in Dallas, September 20, 1903. The funeral was largely attended by prominent Masons from all over the State and by Camps of Confederate Veterans in a body. Addresses were delivered by Dr. Laurence, on behalf of the Confederate Veterans, and by Dr. C. M. Rosser on behalf of the medical profession and the medical college.

Beautiful and touching resolutions were adopted by the Dallas County Medical Society, testifying to the worth and merit of the illustrious physician and to the love, veneration and esteem in which he was universally held.

---

State Health Officer Tabor is "on guard" at Laredo, and has been several weeks, giving his personal and undivided attention to the fever epidemic. Dr. Guiteras, the Marine Hospital expert, is also there and the utmost harmony exists between them, and they work in perfect accord, although Dr. Tabor takes—as he should do—precautions against the spread of the fever by other agencies than the mosquito. To that end he causes the disinfection of everything by sulphur and formaldehyde, and detains all suspects ten days. His course in this and all that he has done is to be commended. The people of Texas may sleep well in the assurance that all that human agency, medical and sanitary skill and watchfulness and zeal in the interest of the protection of the public health can do is being done. Thus far not an exposed person has gotten out of the lines, and no apprehensions are felt that the disease will find a foothold outside of Webb county.

As the daily papers report the situation, any news I could publish as to details, in a monthly, would be stale and no news by the time it reached my readers.

At this writing (October 12th) there have been 210 cases at Laredo and ten deaths, less than five per cent. Either the disease is very mild, the doctors are more successful in treating it than in 1867 and 1878, when I had a tussel with it, or else it is not yellow fever. Although I have not seen a case, I am inclined to the latter belief. I was always skeptical of the diagnosis of the fever of 1898-9, which swept Louisiana, with an average mortality of less than four per cent. Is the game worth the candle? There are several diseases that have a higher mortality, and intercourse with the world is not cut off because thereof. It does look that if the mosquito is the *only* danger—quarantine (non intercourse) is not necessary. But Tabor is taking no chances, and the Texas people will have quarantine.

---

DR. McKNIGHT, Quarantine Officer at Laredo, was stricken down with the fever on the 7th inst., and Dr. Thompson, a volunteer physician from Smithville, was attacked on the 8th. A letter from Dr. Tabor (October 8th), giving this news, says: "We really need immunes. I have applications from physicians to come, and we need two or three more, but can not afford to have non-immunes to come in and get sick." Of Drs. McKnight and Thompson, Dr. Tabor says: "They have been working eighteen to twenty-four hours of each day. Neither is seriously ill at this time."

The "Red-Back" earnestly hopes that they may speedily recover.

---

DR. L. W. COCK, of San Marcos, is in charge of the detention camp at Sanchez, on I. & G. N. R. R., ten miles out of Laredo, and Dr. W. B. Briggs, of Easterly, Secretary of the Great Brazos Valley District Medical Society, is in charge of the one on the Texas-Mexican Railroad between Laredo and Corpus Christi. Both these gentlemen are experienced yellow fever physicians.

## Figures and Tables

**Figure f1:**